# Natural disturbance impacts on ecosystem services and biodiversity in temperate and boreal forests

**DOI:** 10.1111/brv.12193

**Published:** 2015-05-22

**Authors:** Dominik Thom, Rupert Seidl

**Affiliations:** ^1^ Institute of Silviculture, Department of Forest‐ and Soil SciencesUniversity of Natural Resources and Life Sciences (BOKU) ViennaPeter‐Jordan‐Straße 821190ViennaAustria

**Keywords:** fire, wind, bark beetles, disturbance effect, biodiversity, ecosystem services, forest management, salvage logging, prescribed burning, disturbance paradox

## Abstract

In many parts of the world forest disturbance regimes have intensified recently, and future climatic changes are expected to amplify this development further in the coming decades. These changes are increasingly challenging the main objectives of forest ecosystem management, which are to provide ecosystem services sustainably to society and maintain the biological diversity of forests. Yet a comprehensive understanding of how disturbances affect these primary goals of ecosystem management is still lacking. We conducted a global literature review on the impact of three of the most important disturbance agents (fire, wind, and bark beetles) on 13 different ecosystem services and three indicators of biodiversity in forests of the boreal, cool‐ and warm‐temperate biomes. Our objectives were to (i) synthesize the effect of natural disturbances on a wide range of possible objectives of forest management, and (ii) investigate standardized effect sizes of disturbance for selected indicators via a quantitative meta‐analysis. We screened a total of 1958 disturbance studies published between 1981 and 2013, and reviewed 478 in detail. We first investigated the overall effect of disturbances on individual ecosystem services and indicators of biodiversity by means of independence tests, and subsequently examined the effect size of disturbances on indicators of carbon storage and biodiversity by means of regression analysis. Additionally, we investigated the effect of commonly used approaches of disturbance management, i.e. salvage logging and prescribed burning. We found that disturbance impacts on ecosystem services are generally negative, an effect that was supported for all categories of ecosystem services, i.e. supporting, provisioning, regulating, and cultural services (P < 0.001). Indicators of biodiversity, i.e. species richness, habitat quality and diversity indices, on the other hand were found to be influenced positively by disturbance (P < 0.001). Our analyses thus reveal a ‘disturbance paradox’, documenting that disturbances can put ecosystem services at risk while simultaneously facilitating biodiversity. A detailed investigation of disturbance effect sizes on carbon storage and biodiversity further underlined these divergent effects of disturbance. While a disturbance event on average causes a decrease in total ecosystem carbon by 38.5% (standardized coefficient for stand‐replacing disturbance), it on average increases overall species richness by 35.6%. Disturbance‐management approaches such as salvage logging and prescribed burning were neither found significantly to mitigate negative effects on ecosystem services nor to enhance positive effects on biodiversity, and thus were not found to alleviate the disturbance paradox. Considering that climate change is expected to intensify natural disturbance regimes, our results indicate that biodiversity will generally benefit from such changes while a sustainable provisioning of ecosystem services might come increasingly under pressure. This underlines that disturbance risk and resilience require increased attention in ecosystem management in the future, and that new approaches to addressing the disturbance paradox in management are needed.

## I. INTRODUCTION

In recent decades, forest disturbance regimes have intensified in many parts of the world (Chapin *et al*., 2000; Schelhaas, Nabuurs & Schuck, 2003; Balshi *et al*., 2007; Gardiner *et al*., 2010). The frequency of large wildfires in western North America has, for instance, increased by nearly four times in the period 1987–2003 compared to the average for 1970–1986 (Westerling *et al*., 2006), while at the same time bark beetle damage has reached unprecedented levels (Meddens, Hicke & Ferguson, 2012). A similar trend is evident for wildfire, windthrow, and bark beetles in Europe (Schelhaas *et al*., 2003; Seidl *et al.,* 2014). This trend is likely to continue in the future as a result of the climatic changes expected for the coming decades (Seidl, Schelhaas & Lexer, 2011b; Li *et al*., 2013; Reichstein *et al*., 2013; Temperli, Bugmann & Elkin, 2013; Seidl *et al.,* 2014). In many areas, changes in the disturbance regime (i.e. in the distinctive type, size, severity, and frequency of disturbance over extended spatio‐temporal scales) are expected to be among the most severe climate change impacts on forest ecosystems (Lindner *et al*., 2010; Turner, 2010). Disturbances are important natural drivers of forest ecosystem dynamics (Franklin *et al*., 2002; Kuuluvainen & Aakala, 2011), and strongly modulate the structure and functioning of forest ecosystems (Weber & Flannigan, 1997; Turner, 2010). Changing disturbance regimes might thus considerably alter forest ecosystems, with potentially far‐reaching impacts on their biological diversity and capacity to provide ecosystem services to society.

With the aim to provide ecosystem services to society while fostering biodiversity, ecosystem management requires a comprehensive understanding of the impacts of natural disturbances. Notwithstanding this high relevance, natural disturbances have hitherto been discussed inconclusively in the context of ecosystem management, with views and recommendations ranging from strict avoidance of disturbance (due to negative effects on selected ecosystem services) to emulating disturbance in management (to utilize their beneficial effects on biodiversity). On the one hand, substantial efforts are undertaken in research and management to quantify disturbance risk, with the aim to minimize their negative impacts through increasing the resistance of forests to disturbances (e.g. Jactel *et al*., 2009; Overbeck & Schmidt, 2012). Measures such as fostering individual‐tree stability through thinning (Schelhaas, 2008), adapting landscape‐scale harvesting patterns to disturbance risk [e.g. stand edges *versus* the main wind direction (Byrne & Mitchell, 2013)], and choosing a rotation period that balances disturbance risk with economic considerations (Loisel, 2014) have long been practiced in forestry in order to avoid disturbance‐related losses particularly with regard to timber production. On the other hand, with the advent of science‐based ecosystem management and a growing understanding of the integral role of disturbances in natural forest ecosystem dynamics, mimicking natural disturbance regimes to foster elemental processes of ecosystem dynamics is increasingly advocated (e.g. Toivanen & Kotiaho, 2007; Newton *et al*., 2011). Hypothesizing a positive effect of disturbances on biodiversity and acknowledging their role in creating keystone habitats within forested landscapes, these ideas view disturbances as inherently positive. In human‐altered boreal forest ecosystems, for instance, where fire is the major natural disturbance agent, there are suggestions for the application of prescribed burning as a measure to restore natural forest conditions (Bergeron *et al*., 2002; Toivanen & Kotiaho, 2007; Olsson & Jonsson, 2010). In wind‐ and bark beetle‐dominated disturbance regimes the creation of gaps of various sizes and shapes is recommended to mimic natural disturbance regimes and stimulate biodiversity (Franklin *et al*., 2002; Seymour, White & DeMaynadier, 2002; Kern *et al*., 2014).

The valuation of disturbances and their role in management thus seems to vary strongly with the particular objective considered (e.g. biodiversity conservation *versus* timber production). However, only a small proportion of forests serve a sole objective: only about 5% of the world's forests are strict reserves for the conservation of biodiversity (Hoekstra *et al*., 2005), while a similar fraction are designated plantations for the production of wood and biomass (Carnus *et al*., 2006). The large majority of forest landscapes need to fulfill a multitude of functions and services simultaneously, including but not limited to serving as habitat, protecting the soil from erosion, producing timber and biomass, storing carbon, etc. In such situations where multiple objectives need to be met within a forest landscape, disturbances can be expected to have both positive and negative impacts on possible objectives of ecosystem management (see e.g. Huston & Marland, 2003), a hypothesis that we here refer to as the ‘disturbance paradox’. Considering that not only disturbances have increased recently but also the range and demand for societally relevant ecosystem services has been growing steadily in recent decades, we estimate that addressing this paradox will be a key challenge for future forest ecosystem management.

Here we attempt to describe and quantify the various effects of natural disturbances in a literature review and meta‐analysis of disturbance impacts at the global scale. In particular, we examine the effects of three of the most detrimental disturbance agents globally [i.e. fire, wind, and bark beetles (FAO, 2010)], focusing on forest ecosystems of the boreal and temperate biomes, a forest area of approximately 13.5 million km^2^ (Hansen, Stehman & Potapov, 2010). Acknowledging the growing societal importance of a variety of different ecosystem services we not only survey disturbance impacts on traditionally important forest goods (such as timber production) but also include a total of 13 different ecosystem services from all four categories distinguished by the Millennium Ecosystem Assessment in our analysis: provisioning, supporting, regulating, and cultural services (MEA, 2005). Furthermore, we also investigated disturbance impact on three important indicators of biodiversity. Our overall objectives were (*i*) to synthesize the effect of natural disturbances on a wide range of possible objectives of forest ecosystem management, and (*ii*) to investigate standardized effect sizes of disturbance impacts for selected indicators *via* a quantitative meta‐analysis. Based on these analyses we discuss pathways to addressing disturbances in ecosystem management in the particular context of changing disturbance regimes.

## II. MATERIALS AND METHODS

### (1) Literature review

We searched the literature for studies on disturbance by fire, wind and bark beetles, and their impacts on ecosystem services as defined by the Millennium Ecosystem Assessment (MEA, 2005), as well as their effects on biodiversity, focusing on species richness and habitat quality as well as on indices of diversity (e.g. Shannon‐Index, Simpson‐Index, etc.). We restricted our literature review to boreal and temperate forest ecosystems as subtropical and tropical forests differ considerably in ecological processes and anthropogenic impacts. In particular, extratropical forests are generally less diverse than tropical forests, and share a common set of genera as well as drivers of forest dynamics (e.g. temperature) (Thomas & MacLellan, 2002). Furthermore, land‐use history and recent management differ strongly between tropical and extratropical regions, with a long history of intensive human use and several decades of sustainable management in the temperate and boreal zone (Siry, Cubbage & Ahmed, 2005; Canadell & Raupach, 2008). Focusing solely on the boreal and temperate subset of the literature controlled for these broad differences in our analysis, and thus increased the inferential potential with regard to disturbance effects. The literature search was performed using the *Scopus* database (SciVerse Scopus, 2013), and the cutoff date for the inclusion of publications was June 6th, 2013. The search terms and synonyms used are listed as supporting online information in Appendix S1. In total, 1958 papers were identified for screening. From this overall body of literature, reviews and syntheses were excluded in order to avoid double counting and the potential transfer of artifacts or errors from one review to the next (Whittaker, 2010). Furthermore, we excluded articles which did not compare disturbed forests with long‐lasting undisturbed ‘control’ sites. Depending on the study scale and context, either the state before a disturbance, an undisturbed reference, or an assumption about an equilibrium condition was assumed as a reference to determine the disturbance effect. From the 1958 papers screened initially 478 were selected for further analysis. For each of these studies we collected information on geographical location, spatial and temporal scales, assessment methodologies and management treatments (Tables 1 and 2, see online Appendix S2). We furthermore recorded whether the reported disturbance effect is related to single or multiple disturbance events (i.e. disturbance regime). If studies included expert opinions on certain disturbance effects they were initially included in our database, but were subsequently omitted from quantitative analyses. We allowed multiple entries per study, for instance if a study examined more than one disturbance agent, ecosystem service or biodiversity indicator. Furthermore, considering that ecological effects can change over time, we also recorded the temporal time frame for every study. In order to alleviate potential autocorrelation issues, effects were grouped into four different time horizons (i.e. short term: 1–5 years, mid term: 6–25 years, long term: 26–100 years, very long term: >100 years). The final database for analysis contained 887 entries of disturbance effects on ecosystem services and biodiversity.

**Table 1:** Geographic distribution of observations (N = 887) of disturbance impacts on ecosystem services and biodiversity reported in 478 peer‐reviewed publications included in the analysis

		Disturbance agent		
Biome	Continent	Fire	Wind	Bark beetles
Boreal	Africa	0	0	0
	Asia	11	1	0
	Europe	28	23	3
	North America	221	24	30
	South America	0	0	0
	Australasia	0	0	0
Cool temperate	Africa	0	0	0
	Asia	2	10	0
	Europe	54	38	11
	North America	198	25	18
	South America	9	0	0
	Australasia	28	6	0
Warm temperate	Africa	2	0	0
	Asia	10	0	0
	Europe	33	0	0
	North America	55	18	0
	South America	2	0	0
	Australasia	24	1	0
Total		677	146	62

Note that two observations addressing fire and wind impact, respectively, at the global scale, are not included.

**Table 2:** Assessment methodology and focal scale of observations (N = 887) regarding disturbance impacts on ecosystem services and biodiversity reported in 478 peer‐reviewed publications included in the analysis

		Assessment methodology					
Temporal scale	Spatial scale	Empirical	Remote sensing	Simulation	Questionnaire	Expert opinion	Mixed
Short term (1–5 years)	Stand	237	1	12	0	14	1
	Patch	23	0	2	0	0	0
	Landscape	28	0	5	2	14	3
	Region	6	2	24	0	4	2
	Global	0	0	0	0	0	0
Mid term (6–25 years)	Stand	117	0	16	0	7	0
	Patch	16	0	2	0	3	0
	Landscape	12	0	9	0	8	2
	Region	5	10	23	1	3	1
	Global	0	0	0	0	0	0
Long term (26–100 years)	Stand	50	0	12	0	6	0
	Patch	5	0	2	0	3	0
	Landscape	4	0	11	0	4	0
	Region	1	1	24	1	10	0
	Global	0	0	2	0	0	0
Very long term (>100 years)	Stand	22	0	6	0	8	0
	Patch	1	0	2	0	2	0
	Landscape	4	0	11	0	16	0
	Region	4	0	14	0	17	0
	Global	0	0	0	0	0	0
NA	Stand	0	0	0	0	2	0
	Patch	0	0	0	0	1	0
	Landscape	3	0	0	0	10	0
	Region	0	0	3	4	5	0
	Global	0	0	0	0	0	0
	NA	0	0	0	0	1	0
Total		538	14	180	8	138	9

Stand: 1–10 ha, patch: 11–100 ha, landscape: 101–100000 ha, region: >100000 ha. NA: undefined temporal or spatial scale.

### (2) Analysis

We analysed our literature‐derived database of disturbance effects in two steps. First, we assessed the disturbance effect on indicators of ecosystem services and biodiversity. To that end, a descriptive classification of the disturbance impact was made based on the findings reported in the literature (i.e. negative, neutral, mixed, or positive impact of disturbance on the respective indicator). This classification allowed us to synthesize results consistently from different methodological approaches. It furthermore enabled a comparison of disturbance impacts between ecosystem services measured on different scales (e.g. recreational value *versus* carbon storage in a forest landscape), as well as between the impacts on ecosystem services and biodiversity. Initially, we tested whether the observed distribution of studies over response categories differed significantly from a random distribution, i.e. we assessed whether a significant disturbance effect can be established from the literature. Subsequently, we tested for differences in disturbance impact among agents, biomes, and study approaches, evaluating the variation of disturbance impacts with these categories. In an attempt to confirm or reject the hypothesized diverging impacts of disturbance on criteria of relevance for ecosystem management (disturbance paradox hypothesis) we also tested whether disturbance impacts differ between indicators of ecosystem services and biodiversity. Another controversial issue in the context of disturbance management is the effect of salvage harvesting after disturbance, i.e. partial or complete removal of disturbance‐killed trees from a site (Donato *et al*., 2006; Lindenmayer, Burton & Franklin, 2008; Thorn *et al*., 2014). We thus also tested the hypothesis that disturbance effects after salvage differ significantly from unsalvaged conditions. Finally, we also compared impacts of prescribed burning to those of wildfires, in order to test for differences in disturbance impacts from intended and unintended fires. All these tests were conducted using independence tests, a powerful, permutation‐based approach to test the null hypothesis that two variables (measured on arbitrary scales) are independent of each other (Hothorn *et al*., 2008), using the package coin (Hothorn *et al*., 2013) within the R language and environment for statistical computing (R Development Core Team, 2014).

In a second step, in order to determine effect size, we conducted a meta‐analysis based on quantitative information on disturbance impact for two particularly well‐researched criteria: biodiversity and carbon storage. For biodiversity, we analysed disturbance‐induced changes in species richness (*S*′, *N* = 57) and species entropy (*H*′, *N* = 28), the latter represented by the Shannon‐Index of diversity. Due to the limited sample size further subdivision into the effects of disturbance on specific taxonomic groups was not possible. With regard to carbon storage, we distinguished between disturbance effects on total ecosystem carbon (TEC, *N* = 27), aboveground live carbon (ALC, *N* = 38), dead aboveground carbon (DAC, *N* = 25), and soil organic carbon (SOC, *N* = 39) in our meta‐analysis. For all variables the effect size was calculated as the per cent change induced by disturbance relative to the reference condition (control). Only entries from single disturbance events without subsequent salvage logging were considered in this second analysis step. We used multiple linear regression analysis to examine the size and statistical significance of disturbance effects on indicators of carbon storage and biodiversity. To generalize the disturbance regime and allow a comparison across studies we used time since disturbance (in years) and disturbance severity (i.e. proportion of timber volume, basal area, or forest area affected by disturbance, using a scale of 0–1) as covariates in the analysis. These parameters were recently used by Miller, Roxburgh & Shea (2011) in an attempt to generalize disturbance effects on diversity. We analysed the residuals of our regression models for trends as well as for temporal autocorrelation (using a Durbin–Watson test), and found support for the assumptions of homoscedasticity and independence. From these regression models we analysed both the intercepts (i.e. the standardized effect at fixed severity and time since disturbance) and slopes (i.e. how the disturbance effect changes with time and severity). To aid the interpretation of the former we transformed severity to 1–severity in our analysis, making the intercept a standardized effect of 100% severity. Additionally, we fitted multiple linear regression models with disturbance agents and biomes as covariates in order to test for the generality of our findings across agents and geographical locations.

## III. RESULTS

### (1) Disturbance effects on ecosystem services and biodiversity

**Figure 1:**
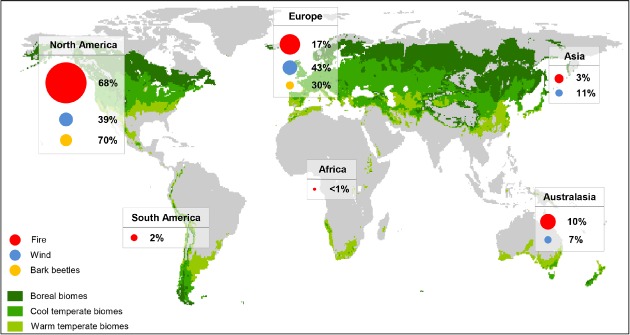
Geographical distribution of papers addressing the impacts of fire (red, comprising wildfire and prescribed burning), wind (blue) and bark beetles (orange) on ecosystem services and biodiversity. The size of the circles represents the number of peer‐reviewed papers per agent and region, while percentages indicate the relative share of disturbance agents per continent. The focal areas of our analysis were the boreal, cool‐ and warm‐temperate biomes as defined by Holdridge (1947, modified using World Clim data), illustrated here in different shades of green.

Overall, 478 studies from the boreal (34.9%), cool (47.1%) and warm temperate (18.0%) biomes addressing effects of disturbances on forest ecosystems were reviewed. The overwhelming majority of articles originated from North America (63.8%), followed by Europe (21.3%) and Australasia (8.8%) (Fig. 1, Table 1). With regard to disturbance agents the effects of forest fires were addressed most frequently (78.0%), while only 15.4% of studies investigated impacts of wind and 6.6% of bark beetles. 60.9% of the research results compiled in our database were empirical, while 19.3% were based on expert opinion, 16.0% derived from simulation studies, and the remaining 3.8% either investigations based on remote sensing, public questionnaires or a combination of different approaches (Table 2). Studies from recent years were overrepresented in our database, with publications on disturbance impact increasing at a rate of approximately 3.1 papers per year between 1996 and 2012 (before 1996 the number of studies was sparse and irregular). This rate of increase of +11.9% year^−1^ is considerably higher than that of the general literature on, e.g. ecosystem management, which was +7.0% over the same period (Seidl, 2014).

**Figure 2:**
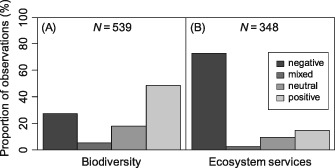
Disturbance effects on (A) biodiversity and (B) ecosystem services. N indicates the number of observations in our database of disturbance effects synthesized from 478 peer‐reviewed articles.

**Figure 3:**
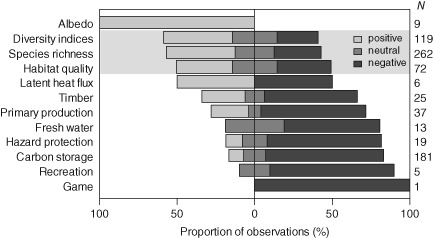
Disturbance effects on indicators of ecosystem services and biodiversity (shaded). Bars show the distribution of positive, neutral and negative disturbance effects per indicator; N denotes the total number of observations. Note that neutral and mixed effects were subsumed under the neutral category here, and that findings based on expert opinions were excluded.

Overall, there is strong evidence for a distinct impact of disturbances on criteria relevant to ecosystem management, with only 19.3% of entries in our database showing no or mixed effects of disturbance. The fact that in our sample of the literature negative impacts (45.1%) and positive effects (35.6%) were nearly equally distributed confirms the hypothesized disturbance paradox in ecosystem management. These divergent impacts are primarily driven by the disparity of disturbance effects on biodiversity and ecosystem services (Fig. 2). We found that all ecosystem service categories [i.e. supporting, provisioning, regulating and cultural services (see online Fig. S1)] were affected predominately negatively by disturbance (*P* < 0.001). At the level of individual ecosystem service indicators, the only investigated aspect that was positively influenced was albedo (Fig. 3), as related to the climate change mitigation function of forest ecosystems (Jin *et al*., 2012). Timber and primary production, fresh‐water provisioning as well as protection against gravitational natural hazards were found to be predominately negatively affected by disturbances. Moreover, the large majority of studies reported a negative disturbance impact on carbon storage, mainly due to a reduction of live biomass in the ecosystem. However, there were also some examples of a positive disturbance effect on carbon storage: in a boreal forest ecosystem in Ontario, ALC peaked 92 years after disturbance then declined to a significantly lower level during the following decades, stabilizing 140 years after disturbance (Seedre & Chen, 2010). For the same forest, SOC peaked between 29 and 140 years after disturbance, before decreasing by approximately one‐third over the next 63 years (Chen & Shrestha, 2012). This suggests that not only direct disturbance‐related C losses in ALC but also the enhanced growth of a regenerating forest as well as the rate of decomposition of dead organic matter need to be considered for a comprehensive assessment of disturbance effects on forest C budgets. Overall, however, 96.3% of 27 observations on C cycle impacts indicated a negative effect of disturbances on TEC.

By contrast, we found an overall positive effect of disturbances on biodiversity (*P* < 0.001). Species richness, habitat quality, and diversity indices were equally positively affected by disturbances. However, the disturbance effect is less consistent for biodiversity than for many ecosystem service indicators, and a number of studies also report negative impacts of disturbances on the indicators of biodiversity investigated here. Hingston & Grove (2010), for example, reported reduced bird species richness in Tasmanian lowland wet eucalypt forests during the first 50 years after wildfire. By contrast, Klaus *et al*. (2010) found a positive effect of fire on the number of bird species in southern Appalachian upland forests. This illustrates that some species groups might react differently to disturbances depending on the context and specific ecosystem investigated. Also belowground diversity is affected by disturbances, yet dedicated studies are still rare to date. Negative impacts on earthworm biomass and diversity at sites with uprooted trees were reported from areas as different as Belgium and northern Iran (Nachtergale *et al*., 2002; Kooch & Hosseini, 2010). Another belowground species group that was reported to be negatively affected by windthrow (salvaged) and fire disturbance was Oribatida in the Slovakian High Tatra Mountains (Lóšková *et al*., 2013). However, a positive impact of fire was reported on soil collembolan diversity in a northern hardwood forest (Huebner, Lindo & Lechowicz, 2012) as well as on soil microbial communities in Spain (Fontúrbel *et al*., 2012), indicating that disturbances can have both positive and negative impacts on soil diversity. Overall, however, 73.1, 69.8 and 65.3% of studies reported either a positive or neutral response of diversity, species richness and habitat quality, respectively, to disturbance.

At the level of different disturbance agents we found no support for significant differences between the effects of fire, wind, and bark beetles on indicators of biodiversity. With regard to ecosystem services, however, the impacts of fire differed significantly from those of wind and bark beetles (*P* < 0.001 and *P* = 0.006, respectively), with the latter agents being more frequently reported to have no influence on ecosystem services. This indicates that bottom‐up disturbances such as fire (i.e. susceptibility decreasing with tree size and/or age) might have different impacts than top‐down disturbances such as wind and bark beetles (where susceptibility increases with tree size and/or age). Differences in disturbance impacts between biomes were evident in our data: the effect of disturbances on ecosystem services differed among the boreal and temperate biomes (*P* < 0.001 and *P* = 0.005 for cool‐ and warm‐temperate biomes, respectively), while boreal and cool‐temperate biomes differed with regard to disturbance impacts on biodiversity (*P* = 0.022). Generally, disturbance effects were least distinctive in the boreal biome, with negative disturbance impacts on ecosystem services more pronounced in the temperate biomes compared to boreal ecosystems. However, disturbances also had a stronger positive effect on biodiversity in the cool‐temperate biome than in the boreal biome.

By comparing results across different types of methodologies, e.g. simulation studies *versus* empirical approaches, we found some noteworthy deviations from the null hypothesis of consistent disturbance impacts across study methods. Concerning the impacts of disturbances on ecosystem services we found a significant difference between empirical studies and simulation studies (*P* = 0.030) as well as an indication for differences between empirical studies and expert opinions (*P* = 0.057), with simulation studies and experts reporting a stronger negative effect than empirical analyses. With regard to the effects of biodiversity, we found that both simulation studies (*P* = 0.007) and expert opinions (*P* < 0.001) differed significantly from empirical studies. Here, our data indicate that simulation studies underestimate the positive effects of disturbance on biodiversity compared to empirical analyses, while experts overestimate this positive effect. It is also interesting to note that neutral effects (i.e. no disturbance impact on biodiversity) were more commonly reported in empirical studies than in any other methodological approach.

### (2) The effect of salvage logging and prescribed burning

We tested whether the reported disturbance impacts of prescribed burning differed relative to those of wildfires, hypothesizing that controlled burns will have fewer negative effects on ecosystem service provisioning. We found no support for this hypothesis: prescribed burns were more frequently reported to have a negative impact on ecosystem services than wildfires (*P* < 0.001). Yet, this result must be interpreted with caution as it is based only on a small sample of studies for the effect of prescribed burning (*N* = 13). With regard to the predominately positive effects of fire on indicators of biodiversity, prescribed burns did not differ significantly from wildfires (*P* = 0.413).

Another frequently discussed management intervention in the context of disturbance management is salvage logging. Based on previous findings, we hypothesized a negative impact of salvage logging on biodiversity (Lindenmayer *et al*., 2008). Although a slight trend was evident in our data (i.e. the positive disturbance effect on biodiversity indicators was more pronounced for non‐salvaged forests), it was not significant in our comparison of 38 observations on salvage logging with 145 observations of unsalvaged disturbance effects (*P* = 0.205). Moreover, with regard to the impact on ecosystem services no significant differences between salvaged and unsalvaged studies were found (*P* = 0.168), however the data reveal a negative trend for salvaged forests.

### (3) The size of disturbance effects on biodiversity and forest carbon storage

Disturbance effects on forest ecosystems differ greatly with disturbance severity and time since disturbance, which is why we studied effect sizes using these two variables as covariates. Time since disturbance significantly explained disturbance effects for all investigated carbon compartments (Table 3). Effects on ALC and DAC were particularly strongly related to this variable, and differences to undisturbed conditions (−91.3 and +155.5% in the first year after disturbance for ALC and DAC, respectively) decreased by +0.6% (ALC) and −1.4% (DAC) on average with every passing year following disturbance. Disturbance severity was not significant in any model, but was retained in the analysis due to its ecological relevance (see also Miller *et al*., 2011). While the analysis of disturbance impacts on indicators of C storage yielded acceptable coefficients of determination (*R* ^2^ from 0.736 to 0.124), the explanatory value of disturbance regime covariates was poor with regard to species richness and entropy. Neither species richness nor entropy was found to differ significantly with time since disturbance and disturbance severity. Tests for differences between agents and biomes overall supported a common global meta‐analysis under consideration of disturbance regime covariates for both response variables (data not shown).

**Table 3:** Meta‐analysis (multiple linear regression) of disturbance effects on indicators of carbon and biodiversity (response variables) and their relation to covariates describing the disturbance regime

	Time since disturbance		1–severity		
Indicator	Coefficient	*P*‐value	Coefficient	*P*‐value	*R* ^2^
ALC	0.606	<0.001	33.461	0.064	0.736
TEC	0.192	0.006	12.860	0.361	0.280
DAC	−1.435	0.014	−477.129	0.200	0.258
SOC	0.260	0.042	−9.075	0.792	0.124
*S*′	−0.307	0.291	−19.400	0.576	0.022
*H*′	−2.608	0.589	−175.386	0.555	0.020

ALC, aboveground live carbon; TEC, total ecosystem carbon; DAC, dead aboveground carbon; SOC, soil organic carbon; *S*′, species richness; *H*′, species entropy (Shannon‐Index).

**Figure 4:**
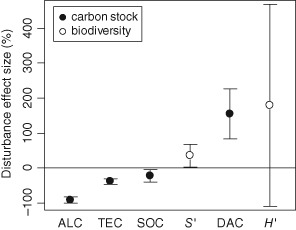
Standardized disturbance effect size (i.e. per cent disturbance‐induced change relative to reference condition) for indicators of carbon stock (filled symbols) and biodiversity (open symbols). Values are standardized coefficients for a disturbance severity level of 100%, and whiskers denote the 95% confidence interval. ALC, aboveground live carbon; TEC, total ecosystem carbon; SOC, soil organic carbon; DAC, dead aboveground carbon; S′: species richness; H′, species entropy (Shannon‐Index).

The analysis of the standardized disturbance effect (i.e. the calculated impact for a year of an event with 100% severity) showed that indicators of biodiversity as well as deadwood C stocks increased with disturbance, while aboveground and soil carbon stocks decreased (Fig. 4). The mean ± 95% C.I. standardized effect of disturbance on total ecosystem carbon was −38.5 ± 8.3% (*P* < 0.001), while species richness was significantly increased by +35.6 ± 32.3% (*P* = 0.035).

## IV. Discussion

### (1) What we know about disturbance impacts on forest ecosystems

We investigated disturbance effects of fire, wind, and bark beetles in a search for general differences in disturbance impacts on ecosystem services and biodiversity. The large number of studies available for analysis not only indicates the importance of disturbance impacts to forest ecosystems, but also provides a suitable basis for a global synthesis on disturbance effects. The increasing number of publications over time may represent a response of the scientific community to the increase in disturbance frequency observed in recent decades (Westerling *et al*., 2006; Seidl *et al.,* 2014), and should imply a growing understanding of disturbance processes. However, while disturbance impacts on biodiversity are increasingly well researched, we found more variability in information on different ecosystem services. While the main focus of the reviewed papers was on regulating services (predominately on C storage as an important mechanism of climate regulation), supporting and provisioning services are less well studied. The disturbance impact on cultural services has barely been assessed to date (see online Fig. S1).

In addition, the information available on disturbance impacts also differs with disturbance agent and region. The impact of fire on biodiversity and ecosystem services is the most intensively studied disturbance agent, reflecting the dominant role of wildfire in disturbance regimes around the globe (e.g. Conard *et al*., 2002; Schelhaas *et al*., 2003; Littell *et al*., 2009; Newton *et al*., 2011; Knox & Clarke, 2012). Regional differences were apparent in our database of published studies on disturbance impacts: Asia, for instance, is underrepresented in our analysis; we found only 10 unique studies on disturbance impacts on biodiversity and 11 on ecosystem services for that continent. However, it has to be noted that not the entire geographic imbalance in disturbance studies is likely to be related to regional differences in scientific understanding of disturbance processes. The main cause of such variation in peer‐reviewed information available from different regions is likely to be the language barrier (Powell, 2012). Differences in local research agendas are also likely to play a role (see e.g. Kajala & Watson, 1997). Nonetheless, we advocate research programs that facilitate a broader study of disturbance effects (geographically as well as in terms of the indicators studied), in order to close some of the remaining gaps in our understanding of the role of disturbances in forest ecosystems.

### (2) Challenges for synthesizing disturbance impacts

One challenge for a global synthesis lies in a comparison of the different methodological approaches used to study disturbance impacts. Simulation approaches appear to underestimate the effect of disturbances on biodiversity perhaps because current disturbance models are rarely able to assess effects on diversity over a broad variety of guilds. Future improvements in simulation modelling should thus aim to capture the multiple impacts of disturbances better on ecosystems and their diversity (see also Seidl *et al*., 2011a). Another interesting finding was that expert knowledge differed significantly from the results of empirical studies. Part of this difference could be explained by expert knowledge being reported for different systems and contexts, i.e. systems and indicators that are less well represented by empirical studies. However, the finding that disturbance impacts estimated by experts are more negative on ecosystem services and more positive on biodiversity than those estimated empirically strongly suggests that expert opinions should be omitted from further quantitative analysis (Whittaker, 2010). It should also be noted that our data – like most published literature reviews – are likely to incorporate a degree of publication bias (Møller & Jennions, 2001), i.e. neutral or mixed effects are likely to be underrepresented.

A second challenge relates to the general ability to synthesize the published literature. Although we found a large number of papers dealing with disturbance impacts on biodiversity and carbon storage, only a limited number (18.4 and 22.4%, respectively) could be used in a quantitative meta‐analysis. In most instances we had to exclude studies due to inconclusive reporting of disturbance severity, or the absence of a proper control, consequently making it impossible to quantify the disturbance effect. We thus call for better reporting, especially the inclusion of summary statistics in publications, and advocate a BACI (before – after, control – impact) design (Stewart‐Oaten, Murdoch & Parker, 1986) to facilitate future syntheses on this topic. The increasing requirement to make the results of studies available upon publication, either as an electronic supplement or in archiving services such as Dryad (http://datadryad.org/) should benefit such syntheses in the future. However, some variation in the choice of an appropriate control to disturbed systems is likely to persist, as, for example, the definition of ‘old‐growth’ conditions often differs regionally. Note also that historic land‐use and management practices may influence reference conditions as well as disturbance drivers and impact (e.g. Carcaillet *et al*., 2009), an aspect that cannot be rectified in a global review and meta‐analysis such as that presented here.

Another difficulty for synthesis and generalization arises from the inherent complexity of disturbance regimes in temperate and boreal forests (see also White & Jentsch, 2001). While we studied three of the most influential disturbance agents globally, other agents of high regional significance were not considered. For example, ash dieback, a disease affecting common ash (*Fraxinus excelsior* L.) trees of all age‐classes, is currently strongly impacting forest ecosystems in many European countries (Halmschlager & Kirisits, 2008; Ogris, Hauptman & Jurc, 2009), but was not included in this analysis. Our first analysis step revealed significant differences in impact among disturbance agents, documenting that the unique ecology of every agent is important for understanding its effects (e.g. which trees are affected and how). In the second step of our analysis we included severity and time since disturbance as covariates in order to generalize across agents in our meta‐analysis. Tests of this generalization assumption show that differences among agents could be explained satisfactorily with these two covariates (data not shown), enabling a statistical analysis across agents and scales. This underlines the potential for a process‐based analysis of disturbance regimes in synthesizing knowledge from individual observations to reach general patterns and principles (Turner *et al*., 1993; White & Jentsch, 2001; Miller *et al*., 2011; Seidl *et al*., 2011a).

However, this ability to generalize might to some degree be attributed to the inclusion of only temperate and boreal forest ecosystems in our data set. Whether the general patterns deduced for these biomes also hold for tropical forests remains to be tested. Martin, Newton & Bullock (2013), conducted a review on the effects of anthropogenic disturbance on carbon stocks and plant diversity for more than 600 secondary forest sites in the tropics. They show that both biodiversity and carbon storage were negatively affected by clearing (a high‐severity disturbance), and took several decades to recover. Assuming that salvage logging after natural disturbance results in an impact comparable to anthropogenic clearing we here find contrasting results for biodiversity effects in temperate and boreal forests: our data suggest a weak positive effect of disturbance on biodiversity (not significantly affected by salvage logging, *P* = 0.205). This indicates that further studies are needed to establish whether the disturbance paradox described here also applies to tropical forests.

The existence and strength of simultaneous positive and negative impacts of disturbances on objectives of ecosystem management, described here as the disturbance paradox, might not only vary geographically but is likely also strongly dependent on the indicators selected for analysis, and hence the local relevance of specific ecosystem services and aspects of biodiversity. Generalist species might, for instance, benefit strongly from disturbance events while specialists and late‐seral species – which are often a priority for conservation – could be negatively affected (Devictor & Robert, 2009). Moreover, disturbances might benefit invasive alien species (see e.g. Crawford *et al*., 2001), widely regarded as negative for biodiversity. Owing to the broad scope of this study such aspects were not explicitly considered in our analysis. They might, however, be of high relevance in local assessments and management decisions, and could thus strongly modify the disturbance paradox, described here based on a global synthesis for boreal and temperate forests. A context‐specific assessment of biodiversity effects at the level of guilds, red‐listed species, and alien/native/endemic species in future studies is thus suggested in order to scrutinize further the generality of the disturbance paradox presented here.

### (3) The disturbance paradox and how to address it in ecosystem management

We found strong evidence for the existence of the disturbance paradox in our global analysis of disturbance impact. Disturbance effects on ecosystem services and biodiversity clearly differ in the published literature, with ecosystem services being overall negatively affected while biodiversity is predominately positively influenced by natural disturbances. Our meta‐analysis of the disturbance effect on species richness and total ecosystem carbon storage aptly illustrates this paradox: while species richness increases by 35.6% on average for a high‐severity disturbance event, a simultaneous loss of 38.5% of total ecosystem carbon storage is to be expected. When management goals are to increase carbon storage while at the same time fostering biological diversity, managers are faced with ambiguity with regard to assessing the impact of a disturbance event, and gauging the implications of future disturbance regimes. Are disturbances to be prevented (as far as possible) to reduce negative impacts on ecosystem services, or are they to be welcomed and incorporated into management due to their positive effects on biodiversity?

While our global study cannot resolve this paradox of ecosystem management – which needs to be addressed in the local context of stakeholder preferences, habitat quality, and other constraints – several interesting insights for disturbance management can be deduced from our analysis. Since negative disturbance impacts on carbon storage are strongly reduced with time since disturbance, but positive effects on biodiversity do not vary significantly over time, our global meta‐analysis suggests that managing for a low‐ to medium‐frequency disturbance regime would result in limited impacts on provisioning services while still benefiting biodiversity. In other words, our data indicate that the disturbance event itself matters for biodiversity, while having enough time between these events ensures recovery of ecosystem services. Albeit not significant in our analysis, the same is true with regard to severity, i.e. moderate‐ or mixed‐severity disturbances (see e.g. Perry *et al*., 2011) are likely to be the best balance between negative effects on ecosystem services and positive effects on biodiversity. Traditional disturbance management approaches such as salvage harvesting and prescribed burning, for instance, are not able to moderate between negative ecosystem service impacts and positive diversity effects according to our analysis. We even found a higher proportion of papers reporting negative effects from prescribed burning on ecosystem services provisioning compared to wildfire. However, due to sample‐size limitations we were not able to analyse these data for differences in effect size, although differences in severity (i.e. mean severity over all studies for prescribed burning = 26.2%, wildfire = 88.1%) suggest a positive effect of prescribed burning (Hurteau & North, 2009; Meigs *et al*., 2009).

Ongoing climatic changes will likely increase disturbance frequency and severity in many parts of the world (Li *et al*., 2013; Temperli *et al*., 2013; Seidl *et al.,* 2014) which – according to our findings – may have negative implications for ecosystem service provisioning. Hence, adaptation of forest ecosystems to such changes in disturbance regime is of great importance in current forest ecosystem management, in order to sustain future ecosystem services provisioning to society. However, as many important drivers of the disturbance regime such as species composition respond to management changes only on time scales of decades to centuries (e.g. Hicke & Jenkins, 2008; Thom *et al*., 2013), such management considerations need to take long lead‐times into account. On the other hand, our analysis indicates that intensifying disturbance regimes may also represent an opportunity to foster biodiversity in forest ecosystems, and might thus to some degree alleviate the ongoing biodiversity crisis (Stuart *et al*., 2004; Thomas *et al*., 2004). In this context it is interesting to note that more diverse ecosystems are often more resistant and resilient to disturbance impacts (Bengtsson *et al*., 2000), so that in the long term disturbance effects on ecosystem services might be buffered by increasing structural and compositional diversity.

## V. CONCLUSIONS

(1) Over the last decades, the number of peer‐reviewed publications on forest disturbances and their effects has increased, mirroring the increasing relevance of disturbance regimes and the changes therein. However, the available literature is heterogeneously distributed over agents and regions, with most studies addressing forests in North America and Europe, and mainly focusing on fire impacts.

(2) Disturbances in forest ecosystems can have both positive and negative impacts on objectives relevant to ecosystem management. We here find that ecosystem services of all four categories defined by the MEA (2005) (provisioning, supporting, regulating, and cultural) are predominately negatively impacted by natural disturbances. Biological diversity, as represented by species richness, habitat quality, and diversity indices is, on the other hand, predominately positively affected by natural disturbances.

(3) In a meta‐analysis we determined that on average a disturbance event decreases total ecosystem carbon by 38.5% (standardized coefficient for a stand‐replacing disturbance event in the year of the disturbance), while species richness increases by on average 35.6%.

(4) For ecosystem management, which aims to provide ecosystem services sustainably to society while preserving and fostering biodiversity, these divergent disturbance impacts present a paradox – they are at the same time risk factors and facilitators of management objectives. Our analysis suggests that measures of disturbance management such as salvage logging and prescribed burning do not significantly moderate these diverging impacts. However, a meta‐analysis of carbon storage (an important regulating service in the context of climate change mitigation) and biodiversity suggests that managing for a disturbance regime of low to medium frequency and severity could limit impacts on ecosystem services while still being beneficial for biodiversity.

(5) Our review suggests that intensifying disturbance regimes under climate change will largely benefit biological diversity of forest ecosystems. Ecosystem services provisioning on the other hand will mostly be negatively impacted by such changes in the disturbance regime. This might require a timely adaptation to changing disturbance regimes in order to provide important ecosystem services sustainably in the future.

## Supporting information


**Fig. S1.** Reported disturbance effects on biodiversity and ecosystem service categories (following the definition of the Millenium Ecosystem Assessment, 2005): (A) biodiversity, (B) supporting services, (C) provisioning services, (D) regulation services and (E) cultural services. N indicates the number of observations.

**Appendix S1.** Indicators of biodiversity and ecosystem services and their respective synonyms used in the literature search.

**Appendix S2.** Database of disturbance impacts on ecosystem services and biodiversity derived from the literature.
