# Supplementary material for: Natural disturbance impacts on ecosystem services and biodiversity in temperate and boreal forests
Source: Biol Rev Camb Philos Soc. 2015 May 22;91(3):760–81. doi: 10.1111/brv.12193 (PMC4898621; doi:10.1111/brv.12193)
Supplement: Supplementary file 1 — Fig. S1. Reported disturbance effects on biodiversity and ecosystem service categories (following the definition of the Millenium Ecosystem Assessment, 2005): (A) biodiversity, (B) supporting services, (C) provisioning services, (D) regulation services and (E) cultural services. N indicates the number of observations. [file BRV-91-760-s003.pdf]

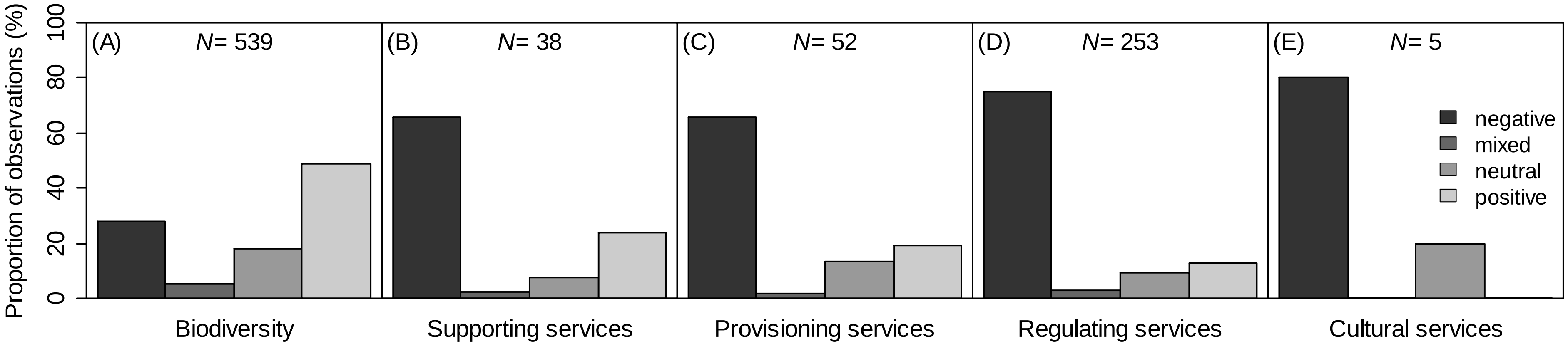

Fig. S1. Disturbance effects on biodiversity and ecosystem service categories (following the definition of the Millennium Ecosystem Assessment, 2005): (A) biodiversity, (B) supporting services, (C) provisioning services, (D) regulation services and (E) cultural services. *N* indicates the number of observations.
