# Supplementary material for: Natural disturbance impacts on ecosystem services and biodiversity in temperate and boreal forests
Source: Biol Rev Camb Philos Soc. 2015 May 22;91(3):760–81. doi: 10.1111/brv.12193 (PMC4898621; doi:10.1111/brv.12193)
Supplement: Supplementary file 2 — Appendix S1. Indicators of biodiversity and ecosystem services and their respective synonyms used in the literature search. [file BRV-91-760-s001.pdf]

Appendix S1. Indicators of biodiversity and ecosystem services and their respective synonyms used in the literature search. The search text combined the indicators and synonyms indicated here with each of the focal disturbance agents (i.e. fire, wind and bark beetles).

Criterion	Indicator	Synonyms
Biodiversity		
Biodiversity	Species richness	
	Habitat	Living space; biotope; structural diversity; species abundance
	Diversity indices	Species diversity; biodiversity; genetic diversity
Supporting services		
Primary production	Primary production	Primary productivity; NPP; GPP
Oxygen production	Air quality	Oxygen
Provisioning services		
Timber	Timber	Biomass production; biomass productivity; timber production; timber productivity; fuelwood
Fresh water	Fresh water	Drinking water; water supply; water provisioning; water purification
Food (plants)	Vegetables	Berries; mushrooms; nuts; herbs
Food (game)	Meat	Animal food; carnal food; fleshy food; hunting; game; venison; deer; elk
Regulating services		
Protection against gravitational natural hazards	Protection against gravitational natural hazards	Rockfall protection; rockslide; avalanche protection; flooding protection; floodwater; flood; mudflow protection; mudslide; landslide; protection against soil erosion; soil loss; erosion
Climate regulation	Carbon storage	Carbon sequestration; net ecosystem productivity; net ecosystem exchange; net ecosystem carbon balance; NEP; NEE; NECB
	Albedo	
	Latent heat flux	
Cultural services		
Recreation	Recreation	Tourism; mountainbiking; hiking; camping; scenic beauty; scenic value
Inspiration	Inspiration	Art; folklore; writing; essay; painting; drawing; sculpture
Intellectual development	Intellectual development	Science; education; cognitive development
